# Sesquiterpene Variation in West Australian Sandalwood (*Santalum spicatum*)

**DOI:** 10.3390/molecules22060940

**Published:** 2017-06-06

**Authors:** Jessie Moniodis, Christopher G. Jones, Michael Renton, Julie A. Plummer, E. Liz Barbour, Emilio L. Ghisalberti, Joerg Bohlmann

**Affiliations:** 1 School of Biological Sciences (M084), University of Western Australia, 35 Stirling Hwy, Crawley, WA 6009, Australia; christophergrahamjones@gmail.com (C.G.J.); michael.renton@uwa.edu.au (M.R.); julie.plummer@uwa.edu.au (J.A.P.); liz.barbour@uwa.edu.au (E.L.B.); 2 School of Chemistry and Biochemistry (M310), University of Western Australia, 35 Stirling Hwy, Crawley, WA 6009, Australia; elg@chem.uwa.edu.au; 3 Michael Smith Laboratories, University of British Columbia, 2185 East Mall, Vancouver, BC V6T1Z4, Canada; bohlmann@msl.ubc.ca; 4 School of Agriculture and Environment, University of Western Australia, 35 Stirling Hwy, Crawley, WA 6009, Australia

**Keywords:** sandalwood, *Santalum spicatum*, *Santalum album*, sesquiterpene fragrance, α-santalol, β-santalol, *E*,*E-*farnesol, naturally occurring chemical variation, chemical diversity

## Abstract

West Australian sandalwood (*Santalum spicatum*) has long been exploited for its fragrant, sesquiterpene-rich heartwood; however sandalwood fragrance qualities vary substantially, which is of interest to the sandalwood industry. We investigated metabolite profiles of trees from the arid northern and southeastern and semi-arid southwestern regions of West Australia for patterns in composition and co-occurrence of sesquiterpenes. Total sesquiterpene content was similar across the entire sample collection; however sesquiterpene composition was highly variable. Northern populations contained the highest levels of desirable fragrance compounds, α- and β-santalol, as did individuals from the southwest. Southeastern populations were higher in *E*,*E*-farnesol, an undesired allergenic constituent, and low in santalols. These trees generally also contained higher levels of α-bisabolol. *E*,*E*-farnesol co-occurred with dendrolasin. Contrasting α-santalol and *E*,*E-*farnesol chemotypes revealed potential for future genetic tree improvement. Although chemical variation was evident both within and among regions, variation was generally lower within regions. Our results showed distinct patterns in chemical diversity of *S. spicatum* across its natural distribution, consistent with earlier investigations into sandalwood population genetics. These results are relevant for plantation tree improvement and conservation efforts.

## 1. Introduction

West Australian sandalwood (*Santalum spicatum*) belongs to the genus of hemi-parasitic *Santalum* (Santalaceae) trees widely exploited for their fragrant heartwood which is used in perfumes, pharmaceuticals, incense and ornamental carvings. The fragrance contained in the heartwood of mature sandalwood trees (>10 years) consists of a complex mixture of sesquiterpenoids, with unique compositions apparent across, and often within species [1,2,3]. Historically, Indian sandalwood (*S. album*) has provided the bulk of sandalwood products; however, *S. spicatum* is frequently used as a supplement to incense feedstock and to a lesser extent as extracted fragrance oil. The international standard for *S. album* oil requires 41%–55% of the sesquiterpene alcohol α-santalol and 16%–24% β-santalol [4,5]. At present, *S. spicatum* extracts do not meet these industry standards for two reasons; the combined santalol content is too low, and levels of *E*,*E-*farnesol, a suspected allergen, are too high [6,7,8]. Extracts of *S. spicatum* are generally considered less valuable than those of *S. album* due to the lower overall content and more variable sesquiterpene composition [6].

*S. spicatum* is traditionally harvested from natural stands of undomestiacted tree populations throughout south-western Australia. To reduce pressure on wild stands, plantations have been established on former grazing and marginal agricultural land. Significant potential exists for the improvement of *S. spicatum* plantations through selection of trees with desired growth and sesquiterpene characteristics. By first quantifying the extent and nature of sesquiterpene variation throughout the tree’s area of distribution, key selection parameters may be specified. Moreover, by better understanding the process of sesquiterpene formation in sandalwood, relevant molecular markers may be developed to speed up the selection process. Continued development of *S. spicatum*, a species naturally adapted to the arid-zone of Western Australia, will further conservation goals as well as improving its commercial potential.

**Figure 1:**
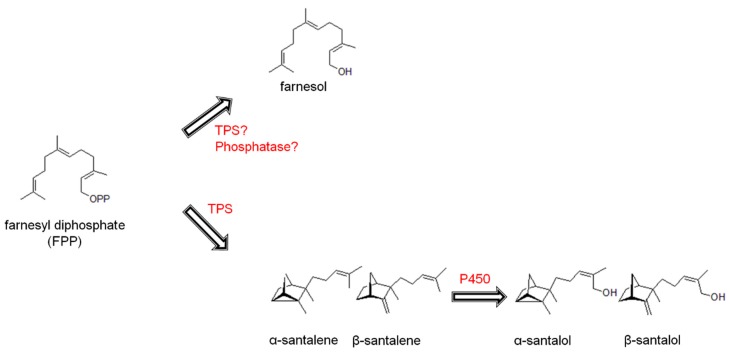
Biosynthesis of major sesquiterpene alcohols in *S. spicatum.* Proposed formation of farnesol from farnesyl-diphosphate (FPP) via a terpene synthase (TPS) or phosphatase activity, and the known pathway for santalol biosynthesis in sandalwood via TPS and cytochrome P450 (P450) activities.

The heartwood extract of *S. spicatum* contains over 100 different sesquiterpenes [9,10,11,12]. This complex mixture of sandalwood sesquiterpenes is likely to serve as protection against wood-rotting fungal pathogens, given its highly-enriched localization specifically in the innermost heartwood [13]. The most abundant compounds of the *S. spicatum* heartwood extract are the sesquiterpene alcohols *E*,*E-*farnesol, α- and β-santalol, lanceol, nuciferol and α-bisabolol, as well as a variety of sesquiterpene olefins such as the santalenes, bergamotene, and several curcumenes [11,12]. Moretta [2] studied the heartwood-cores of 87 *S. spicatum* trees from 12 geographic sites in Western Australia and, in the form of a PhD thesis, reported the levels of α- and β-santalol to vary 3%–67%, and *E*,*E-*farnesol to range 5%–30% across the entire distribution (Figure 1). Although variation in major components has been reported for *S. spicatum,* there have been no further investigations into the sesquiterpene composition across populations within its natural distribution. A more comprehensive evaluation of the extent and nature of variability of wild *S. spicatum* trees is needed to inform efforts to increase total santalol content through selection.

Chemical variation in *S. spicatum* might be explained by genetic differences amongst populations, while the species is also growing across a range of diverse environments. Discrete chemical differences in heartwood composition appear to exist throughout the species’ natural distribution [2] and several populations have been shown to bear strong genetic similarities [14,15]. *S. spicatum* is distributed throughout the semi-arid southern regions (300–600 mm annual precipitation) and arid northern areas of Western Australia (150–300 mm), with large differences in soil moisture levels, soil types, root haustorial connectivity, as well as presence of pests and pathogens [16]. These biotic and abiotic environmental factors may influence the sesquiterpene profile of a tree.

**Figure 2:**
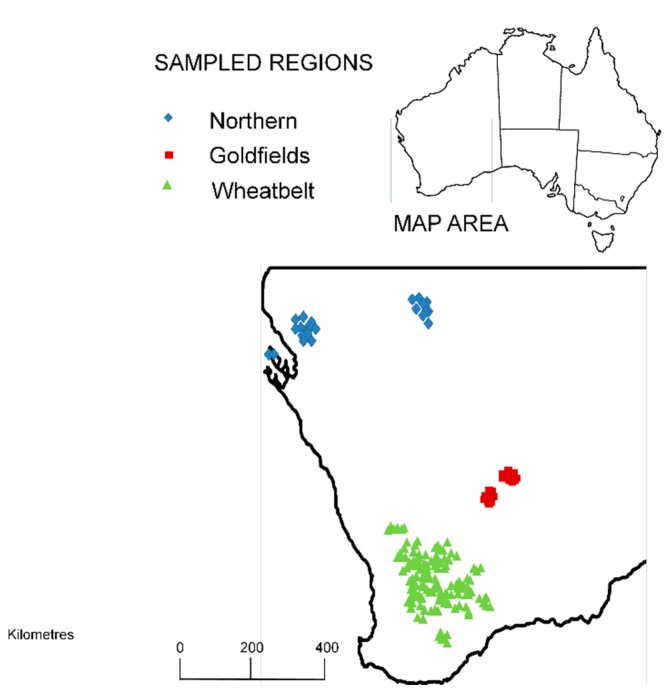
Map of Western Australia showing locations for collection of 194 *S. spicatum* heartwood-cores for chemical analysis.

In this study, the diversity and variation of sesquiterpenes of *S. spicatum* heartwood was characterized and examined for patterns indicative of unique chemotypes. Heartwood core samples were taken from 194 trees across the three main regions where *S. spicatum* naturally occurs. While the majority of samples were taken from the south-west (aka Wheatbelt region) of Western Australia, where a long history of harvesting is known, chemical analysis was extended to populations in the arid north and the south-east (aka Goldfields region) of Western Australia (Figure 2). It was hypothesized that chemically distinct populations of *S. spicatum* would be present, and that the chemical diversity of heartwood sesquiterpene profiles would be lower within populations, while being higher between populations.

## 2. Results and Discussion

### 2.1. Variability of Total Sesquiterpene Content

Total yield of sesquiterpene extracts from heartwood of *S. spicatum* individuals sampled at a stem height of 30 cm above ground was not significantly different across the three regions (Table 1). Trees in the Wheatbelt (3.22 ± 1.08%, *n* = 152), Goldfields (2.40 ± 0.94%, *n* = 19) and north (3.18 ± 1.15%, *n* = 23) contained similar total sesquiterpene contents, with individuals ranging 0.01%–6.41% by dry-weight. High standard deviations suggest yield variation is highest between individuals, rather than among regions. Reported sesquiterpene oil contents are similar to previous studies on mature *S. spicatum* trees from natural stands [2,17]. Tree to tree variation in total terpene concentrations has also been reported in other terpene accumulating species (e.g., Egerton-Warburton et al. [18]). The observed variation in *S. spicatum* can be explained in part by tree maturity, as higher heartwood sesquiterpene concentrations are more common in older trees [6]. Mean tree-age should be lower in more frequently harvested regions. Sandalwood in the Wheatbelt and Goldfields has been more heavily exploited for a longer period of time than those in the North. Biometric parameters such as diameter and tree height or growth rings do not appear to be reliable proxies for tree age in wild stands of *S. spicatum*, given the broad distribution of the species, the wide range of growing conditions and the hemi-parasitic nature of sandalwood [19,20,21]. Other sandalwood species such as *S. austrocaledonicum* have also displayed variation in total sesquiterpene oil yield [3]. Even *S. album*, the terpene extract of which can consistently contain more than 80% α- and β-santalol [22] varies widely in yield from no detectable sesquiterpene oil to nearly 9% by dry weight. Tree age has been reported to be a significant component of yield variation in plantations of *S. album* [1]. However, genetic and environmental factors contributing to heartwood development in general and regulation of sesquiterpene production in particular in any *Santalum* species are poorly understood [23].

### 2.2. Sesquiterpene Profiles and Geographic Variation

**Figure 3:**
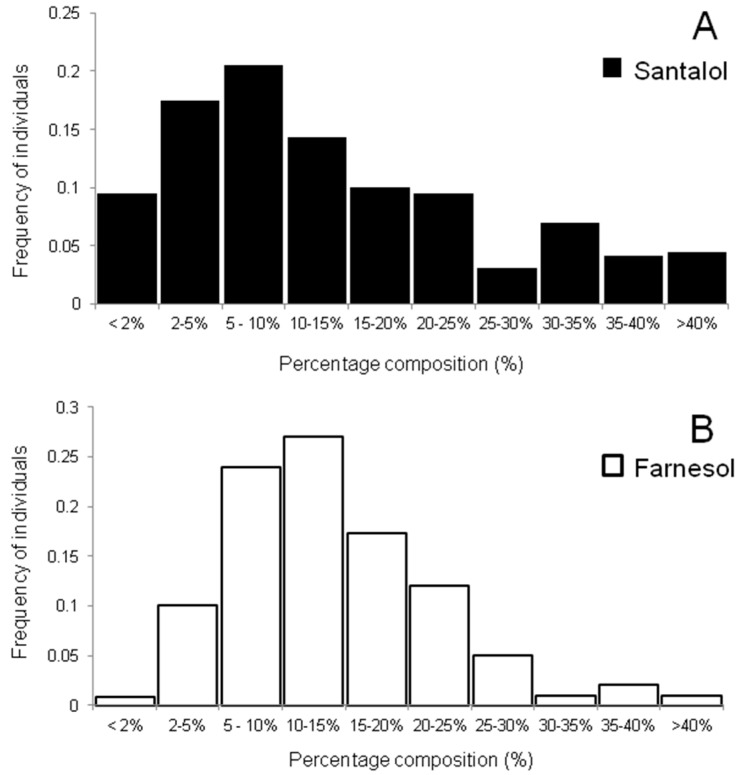
Frequency of *S. spicatum* individuals with different relative amounts of (**A**) α- and β-santalol concentration and (**B**) *E*,*E-*farnesol in heartwood samples of 194 trees growing in natural stands in Western Australia: 152 trees were from the south-west (Wheatbelt), 19 trees from the southeast (Goldfields) and 23 trees from the north regions (Carnarvon and Shark Bay).

The heartwood sesquiterpene composition of *S. spicatum* trees sampled in this study varied both within and between regions (Table 1). The two main sesquiterpene alcohols imparting value to the oil, α- and β-santalol, were found to be highest in trees from the north (33.0 ± 11.6% santalols), followed by the Goldfields (12.5 ± 10.9%). Wheatbelt trees produced extracts with the least amount of santalols (10.3 ± 7.0%). Large standard deviations are indicative of the natural variation in santalol content, which across the three regions ranged 0.8%–55.6% of the total extract. Approximately one quarter of all trees surveyed comprised less than 5% combined α- and β-santalol levels, which is considered very low quality [4] and more than half the trees had extracts with less than 10%–15% combined santalol content (Figure 3). A few individuals (0.5%) had extracts with greater than 50% total santalol content, mainly those from the north. These levels are more similar to those found in *S. album* heartwood. Although the sample size was small for the north (*n* = 23) and the Goldfields regions (*n* = 19), all tree sampling was done over a wide area at each site in an effort to minimize sample selection bias. All individuals from the north contained at least twice as much α- and α- santalol as farnesol in the extract. No individuals in this study reached the ISO standard [5] for *S. album* (41%–55% α-santalol and 16%–24% β-santalol), however several high-quality trees were identified, with one northern tree containing 55.6% combined α- and β-santalol content and less than 5% farnesol. Despite the sesquiterpene composition varying across the natural distribution, the identification of *S. spicatum* trees with high levels of α- and β-santalol and simultaneously low levels of farnesol was important as it indicates the potential to select individual trees with superior fragrance qualities from these populations for future improvement programs.

Trees from the north of Western Australia contained generally lower levels of *E*,*E-*farnesol than trees from the Wheatbelt and Goldfields regions, and variation within regions was also evident. Trees from the Goldfields had on average the highest amounts of *E*,*E-*farnesol (19.4 ± 5.6%) followed by trees from the Wheatbelt (14.4 ± 8.5%), while those in the north had the least (9.1 ± 5.4%). Variation in *E*,*E-*farnesol content across all regions was high, comprising anywhere between 2.0% and 46.2% of the total sesquiterpene profile. A frequency distribution of *E*,*E-*farnesol chemotypes highlights this variation (Figure 3). Ten percent of sampled treed contained high levels of farnesol (>35%) while trees with less than 5% farnesol were similarly infrequent. Some chemotypes of sandalwood appear to exist with high or low levels of *E*,*E-*farnesol, however the bulk of the distribution (more than half of the trees analyzed) contained between 10% and 35% *E*,*E*-farnesol, suggesting a continuous distribution. It is perhaps unsurprising that distinct chemotypes of *S. spicatum* could exist in different regions of Western Australia; Byrne et al. [14] identified that the northern arid populations of *S. spicatum* were genetically distinct from the southern semi-arid populations (including the Wheatbelt). Moretta [2] also suggested chemotypes of *S. spicatum* may exist in geographically distinct pockets of Western Australia. Of the 12 regions sampled, Moretta [2] found *E*,*E-*farnesol to be lowest in the northwest region (Shark Bay) (<5%) and highest (0%–35%) in a south-west population (Katanning) of Western Australia. Additionally, Moretta [2] reported that three populations from the northern parts of Western Australia had the highest average santalol content compared to other regions of the state. Despite limited by small sampling size, these results are in strong agreement with the findings of the present study.

Future efforts into tree improvement of *S. spicatum* for plantations would benefit from an understanding of the genetic and molecular underpinning of the accumulation of *E*,*E*-farnesol and its variation, as it directly impacts on the quality and marketability of the extracted fragrance oil [4,9,11,17]. Moreover, abiotic environmental conditions such as temperature, rainfall and soil structure, as well as biotic factors including tree age, growth rate, neighboring host species and disease prevalence vary widely across sandalwood distribution, and are likely to contribute to variation in sesquiterpene profiles between trees [16]. To date, little research has focused on specific environmental, genetic and physiological factors and their function in heartwood sesquiterpene production largely due to difficulties in maintaining suitable controls in field experiments. Clonally propagated sandalwood would reduce genetic variation and would allow for the testing of environmental factors affecting sesquiterpene production; however, *S. spicatum* is difficult to clonally propagate from somatic tissue [24]. Future research directed towards understanding the genetic and environmental origins of chemical variability within this species will aid tree improvement programs which can assist in the selection of superior genotypes with increased production of α- and β-santalol and conversely, low amounts of *E*,*E-*farnesol.

### 2.3. Variation of Other Sesquiterpene Components

Gas chromatography-mass spectrometry (GC-MS) analysis of *S. spicatum* heartwood revealed 17 major sesquiterpene hydrocarbons and alcohols, which contribute up to 80% of the total sesquiterpene oil composition. Even where total sesquiterpene contents were barely detectable, the main components α- and β-santalene, α- and β-santalol, *E*,*E-*farnesol, denrolasin, *Z*-lanceol, *Z*-nuciferol/*Z*-γ-curcumen-12-ol and *Z*-β-curcumen-12-ol were always present (Table 1). Most of these compounds are also found in other sandalwood species [10]. The apparent fixation of these compounds across different *Santalum* species suggested they may contribute fitness. Conversely, other components such as α-bisabolol, dendrolasin and nerolidol were more variable across sampling locations (Table 1). Mean α-bisabolol content in the north and Wheatbelt (1.50 ± 1.7% and 1.80 ± 3.3% respectively) were markedly lower than in Goldfields trees (9.70 ± 4.9%), suggesting an α-bisabolol chemotype might be present in this region. Trees sampled from the north also contained higher levels of the olefins α- and β-santalene (1.2 ± 0.5%) compared to Wheatbelt (0.5 ± 0.3%) and Goldfields trees (0.2 ± 0.1%). Differences in the relative abundances of the santalenes may be due to differential expression or activity of santalene synthases [25], which produce the santalenes, and cytochromes P450 [23,26], which convert the santalenes to the santalols. Expression of the sanatelene synthase and P450 genes may be developmentally regulated or affected by environmental factors as is known for terpene synthases and terpene converting P450s in other systems [27,28]. Sandalwood may be unusual in this regard as sesquiterpenes appear to be produced, and stored indefinitely, in heartwood xylem. As accumulation of the sesquiterpene heartwood oil generally initiates by 10 years of age and continues for the life of the tree, and little to no seasonal variation is evident [2], differential biosynthesis of individual metabolites may be more subtle than in short-lived species or in species with strong seasonal variation of metabolic activity.

### 2.4. Statistical Analysis of Chemotypes

**Figure 4:**
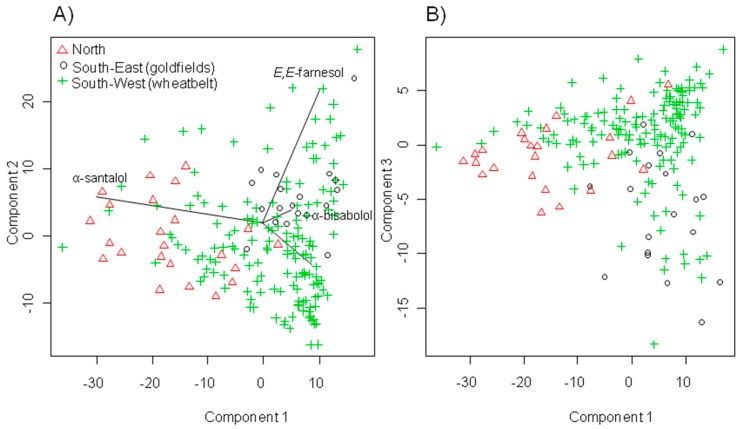
Two dimensional principal component analysis (PCA) ordination scores of *S. spicatum* sesquiterpene samples from the heartwood of 194 trees. Each point represents an individual tree, and points close together are similar in terms of composition. The first three components represent 95% of the total variance: (**A**) represents components 1 and 2 and (**B**) represents components **1** and **3**. Lines indicate ordination scores of the variables used.

Principle component analysis (PCA) indicated variation in the composition of heartwood oil of *S. spicatum*, with a number of santalol and farnesol rich individuals distributed across its natural range The first two principle components, which explained 83% of the variance is shown in a two-dimensional plot (Figure 4a) and indicated that trees within regions tended to share a more similar heartwood chemistry than geographically distant populations, although there were similarities among regions as well, particularly with north and Wheatbelt trees. Overlap in the terpenoid profiles showed that the composition across regions was not sufficiently differentiated based on genetic or geographic differences. PCA revealed high levels of correlation between sesquiterne components, with the first three principle components accounting for 95% of the variance (Figure 4). The analysis indicated that *E*,*E-*farnesol and α-santalol are two largely independent drivers of variability and are the variables mainly responsible for the ordination in the extract of *S. spicatum*. The α-santalol chemotypes with low levels of *E*,*E-*farnesol were mostly represented by trees from the north and several individual from the south-west (Wheatbelt) displayed some remarkable similarities in total sesquiterpene profiles despite their geographic separation. The PCA also suggested that the sampled trees from the south-east (Goldfields) did not contain any individuals with a high α-santalol content, however *E*,*E-*farnesol in addition to α-bisabolol were more important contributors to the total sesquiterpene profile of these trees. These results further support the potential for chemical selection of *S. spicatum* trees for tree improvement programs.

**Figure 5:**
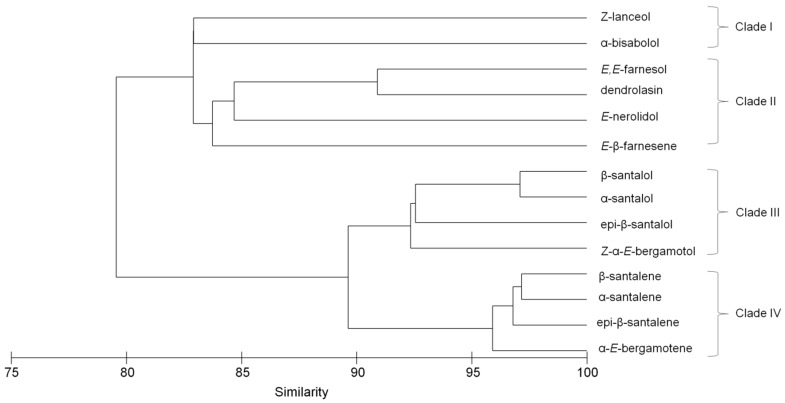
Dendrogram showing co-occurrence patterns of 13 major components using hierarchical cluster analysis from the heartwood extract samples of 194 *S. spicatum* individuals distributed in Western Australia: 152 trees from the south-west (Wheatbelt), 19 trees from the south-east (Goldfields) and 23 trees from the north regions (Carnarvon and Shark Bay).

Variation in terpenoids can be used to some degree to predict pathways of biosynthesis within the plant [1]. In sandalwood, sesquiterpenes are synthesized via terpene synthases [13,28] and cytochrome P450s [23,26]. While some terpene synthases produce a single product, many reported terpene synthase enzymes catalyze the formation of multiple products [26]. A hierarchical cluster analysis identified patterns of accumulation and provided an approximation to the number of different terpene synthase enzymes which may produce the major *S. spicatum* components (Figure 5). The four main clades of the dendrogram indicate at least four separate sets of enzymes may be responsible for producing the major sesquiterpenes and their variations in *S. spicatum*. This is in part confirmed by the knowledge of a previously characterized santalene synthase from *S. spicatum* (*Sspi*SSy) which is responsible for producing the four compounds, α, β-, *epi*-β-santalene and α-*trans*-bergamotene represented in clade IV [28]. In addition, the hydroxylated analogues of the santalenes in clade III of the dendogram, were recently reported to be the products of a cytochrome P450 SaCYP736A167 from *S. album*, which produced α-, β, *epi*-β-santalol, α-*trans*-bergamotol [23]. In *S. spicatum*, the additional characterization of the biosynthetic pathway of *E,E-*farnesol remains an important objective of future work, since this component reduces the market potential of the oil. Clade III of the dendogram contains a cluster of *E*,*E*-farnesol, dendrolasin, *E*-nerolidol and *E*-β-farnesene which are structurally similar (Figure S1, Supplementary Materials). *E*,*E-*farnesol is highly variable in *S. spicatum*, so if a correlation exists between *E*,*E*-farnesol and other structurally similar oil components, a single enzyme might produce both components, stemming from a common intermediate. *E*,*E*-farnesol and dendrolasin showed a strong positive correlation (*r*^2^ = 0.53), suggesting some level of shared biogenesis. Dendrolasin is a furan sesquiterpenoid structurally similar to farnesol, so it is possible that the two compounds share a common origin. Alternatively, *E*,*E-*farnesol may be a minor component of reactions leading to dendrolasin, *E*-nerolidol and *E*-β-farnesene (*P* < 0.05), thus complicating the linear relationship and producing lower coefficients of correlation (Figure S1, Supplementary materials). The possibility for multiple biosynthetic pathways for one component could also help explain some of the quantitative variation that exists in sesquiterpene profiles across the geographic range of *S. spicatum*. Identification of additional pathways is important to understanding total heartwood sesquiterpene production and its regulation in *Santalum* and whether it can be manipulated to increase expression of certain components.

The genetic heritability of various economically important traits (such as sesquiterpene composition, vigor and growth form) has not been examined extensively in *S. spicatum*. Other terpenoid rich plant species such as culinary basil (*Ocimum*, Lamaniaceae) have been successfully bred for desirable chemical characteristics based on studies into chemical composition and field experiments. Like *S. spicatum*, many cultivars of basil vary in their aroma, with chemotypes of citral, eugenol, linalool, methylchavicol and methylcinnamate well represented [29]; and variation of composition under different conditions needed to be taken into consideration when devising selection criteria [30]. Chemical selection and breeding resulted in basil cultivars with up to 15% more methyl cinnamate than the wild types indicative of a high heritability of aroma compound biosynthesis. A similar approach could be tested for *S. spicatum* based on sesquiterpene composition data presented here using seeds sourced from individuals where the desired oil characteristics are evident: high total heartwood sesquiterpene oil content, high levels of α- and β-santalol and low *E*,*E-*farnesol amounts. While breeding cycles will be lengthy, the resulting seedstock would be considerably more valuable. Complementing such efforts, if associations of genetic or genomic markers with terpenoid profiles could be established in future work, the length of breeding cycles could be reduced.

Terpene biosynthesis in *S. spicatum* may be under tight genetic control. Expression and regulation of certain components is likely to be affected by the environment, since inter-population similarities in sesquiterpene profiles were generally evident, but not always the case. Future work should focus on determining the exact genetic and environmental contributors influencing chemotype heritability and further explore the pathways for sesquiterpene biosynthesis*.* This will enable a more comprehensive understanding of factors involved in total sesquiterpene oil production across *Santalum,* and whether this can be manipulated within a tree to increase expression of certain genes or initiate early heartwood-oil development. The future aim is to ultimately reduce the overall domestication time and increase the market potential of sustainable sandalwood production.

## 3. Materials and Methods

### 3.1. Substrates and Reagents

Chemicals, substrates and reagents were purchased from Sigma Aldrich (St. Louis, MO, USA) unless otherwise indicated.

### 3.2. Plant Material Collection

Collection of *S. spicatum* plant material was done in the spring of 2009. Plant material was obtained from trees growing throughout the southern half of Western Australia. Samples collected from the south-west Wheatbelt (*n* = 152), south-east Goldfields (*n* = 19) the Northwest (*n* = 23) (Figure 2). Xylem was collected by drilling into the stems of trees at 30 cm stem height above ground level with a 25 mm drill bit and cordless drill. Only trees with a stem diameter greater than 7 cm were sampled. Wood shavings to be extracted for analysis were air dried in paper bags for two weeks.

### 3.3. S. spicatum Heartwood Extraction and GC-FID and GC-MS

Wood shavings were coarsely ground to homogenize samples; however, grinding was kept to a minimum to prevent volatisation of essential oils. Sesquiterpene rich oil was extracted from ground wood (3–5 g) in 25 mL volumetric flasks using ethanol for 7–14 days. 10 mM isobutyl benzene (IBB) (Sigma Aldrich, St. Louis, MO, USA) was added as an internal standard (IS) for quantification and was selected as it elutes at an earlier retention time to sandalwood components on a DB-Wax column. The extract was separated by gas chromatography (GC) and quantified by flame ionization detection (GC-FID). Identification of components was done using mass spectrometry (MS) (HP6890 Series, Hewlett Packard, Palo Alto, CA, USA), based on comparison of their mass spectra with NIST Libraries as well as by comparison of their retention indices with literature values [12]. An external standard plot using a dilution series of authentic *S. spicatum* oil (Mt. Romance, Albany, WA, Australia) was generated to determine total oil yield from extracts. Relative response factors were determined for α-bisabolol and farnesol standards. To our knowledge, pure standards of other sandalwood oil components are not commercially available. The relative response factor was calculated as:

(1) $$RRF=\frac{area_{IS}\times concentration_{compound}}{concentration_{IS}\times area_{compound}}$$

Gas chromatography conditions were as follows: stationary phase; DB-Wax column, 30 m × 0.25 mm ID × 0.25 µM film thickness (Agilent, Santa Clara, CA, USA). Carrier gas was helium at 1 mL/min. One µL of extract was injected using a split ratio of 10:1. Injector temperature was 200 °C, detector was set to 250 °C. Oven was programmed at 40 °C and held for 1 min, then raised at 3 °C per minute to 220 °C where it was held for a further 25 min. Column conditions for GC-MS were similar to that of GC-FID, except the detector was opened 5 min after injection. Scan mode was used over the range of 41 to 250 *m*/*z*.

### 3.4. Statistical Analysis

Hierarchal cluster analysis was performed to explore co-occurence patterns of major sesquiterpene constituents across the 194 *S. Spicatum* individuals using Primer 6 [31,32]. Data was standardised prior to analysis. A Bray-Curtis similarity was performed for construction of the dendogram using group and single-linkage results (both were comparable). Principal component analysis (PCA) was performed in the R software for statistical computing and graphics [33] using percentage composition data to explore which individuals were similar and different in terms of sesquiterpene composition, investigate correlations in sesquiterpene components, and to identify possible *S. spicatum* chemotypes.

## 4. Conclusions

This study highlighted variations in sandalwood quality (assessed by α- and β-santalol content and *E*,*E-*farnesol) of *S. spicatum* across the semi-arid and arid-regions of Western Australia and showed the existence of different *S. spicatum* chemotypes in three regions of Western Australia: the semi-arid south west Wheatbelt and the arid south-east Goldfields regions (both of which past and current harvesting practices occur) and the north Carnarvon region. The northern trees showed, on average, sesquiterpene profiles of higher quality than trees from the south; however, yields varied within regions rather than across. PCA analysis indicated that α-santalol and *E*,*E-*farnesol chemotypes existed and that it may be possible to select for trees predisposed to increased santalol production. The variation in sesquiterpene oil yield and composition within and across regions could be influenced by a range of factors including age of the trees, growth rate, and environmental variations such as soil composition, host tree availability, rainfall or the presence of pathogens which may stimulate production of certain sesquiterpene components. Tree improvement and better management of natural stands and plantations will be achieved through continued research into genetic and environmental factors which influence heartwood sesquiterpene oil production, as well as factors which might predispose specific chemical phenotypes.

## Figures and Tables

**Table 1:** Sesquiterpene composition of S. spicatum heartwood oil extracts from three different regions of West Australia (Wheatbelt, Goldfields and North).

R.I.		Southwest of W.A. (Wheatbelt)				Southeast of W.A. (Goldfields)				North of W.A. (Carnarvon/Shark Bay)			
Components		Average (%)	Min	Max	SD	Average (%)	Min	Max	SD	Average (%)	Min	Max	SD
*E*,*E*-farnesol	2361	14.4	2.0	46.2	8.5	19.4	9.3	36.5	5.6	9.1	2.1	20.1	5.4
Total α- and β-Santalol		12.5	0.9	54.3	10.9	10.3	0.8	23.5	7.0	33.0	8.7	55.6	11.6
*Z*-nuciferol, *Z*-ϒ-curcumen-12-ol	2520 2525	11.2	3.1	23.3	4.6	8.9	3.6	15.4	3.0	5.9	0.7	19.0	4.7
α-santalol	2346	9.3	0.6	41.3	8.3	7.4	0.5	16.6	5.0	24.8	6.2	38.3	9.2
*Z*-β-curcumen-12-ol	2470	6.9	0.3	17.1	3.5	6.8	0.6	14.0	4.2	6.9	0.3	19.8	5.2
β-santalol	2429	3.2	0.3	13.0	2.7	2.9	0.3	7.0	2.0	8.2	2.5	13.4	2.8
*Z*-α-trans-bergamotol	2358	2.8	0.0	14.0	2.2	2.7	0.2	6.3	1.6	4.2	1.2	7.3	1.9
α-bisabolol	2220	1.8	0.0	18.4	3.3	9.7	2.2	19.0	4.9	1.5	0.1	6.0	1.7
*Z*-lanceol	2496	1.4	0.3	8.7	1.4	5.0	1.5	9.6	2.4	2.3	0.9	6.9	1.4
dendrolasin	1927	1.3	0.2	5.3	0.9	2.3	0.7	5.4	1.2	0.7	0.2	1.5	0.4
epi-β-santalol	2414	0.6	0.0	1.9	0.4	0.5	0.0	1.3	0.4	2.1	0.5	4.8	1.1
α-santalene	1560	0.5	0.0	2.1	0.3	0.2	0.0	0.5	0.1	1.2	0.5	2.3	0.5
trans-nerolidol	2033	0.4	0.0	1.7	0.3	0.6	0.2	1.5	0.4	0.7	0.1	2.2	0.6
β-santalene	1640	0.4	0.0	1.6	0.3	0.1	0.0	0.4	0.1	1.1	0.5	2.0	0.4
*E*-β-farnesene	1760	0.3	0.0	1.2	0.2	0.0	0.0	0.1	0.0	0.0	0.0	0.1	0.0
epi-β-santalene	1621	0.2	0.0	1.1	0.2	0.1	0.0	0.2	0.1	0.7	0.3	1.3	0.3
α-trans-bergamotene	1576	0.2	0.0	0.7	0.1	0.1	0.0	0.1	0.0	0.4	0.2	0.8	0.2
Total		54.8	24.6	81.9	10.3	66.7	51.2	76.0	5.7	69.9	56.7	78.5	6.3
Yield (%)		3.22	0.01	6.41	1.08	2.40	0.58	4.61	0.94	3.58	1.48	5.79	1.15

## Supplementary Materials

The following are available online, Figure S1: The linear correlation between *E*,*E-*farnesol, dendrolasin, *E-*nerolidol and *E-*β-farnesene based on GC-MS analysis 194 heartwood cores of *S. spicatum* trees in natural stands of Western Australia.
