# Supplementary material for: Sesquiterpene Variation in West Australian Sandalwood (Santalum spicatum)
Source: Molecules. 2017 Jun 6;22(6):940. doi: 10.3390/molecules22060940 (PMC6152738; doi:10.3390/molecules22060940)
Supplement: Supplementary file 1 [file molecules-22-00940-s001.pdf]

Supplemental Figure S1

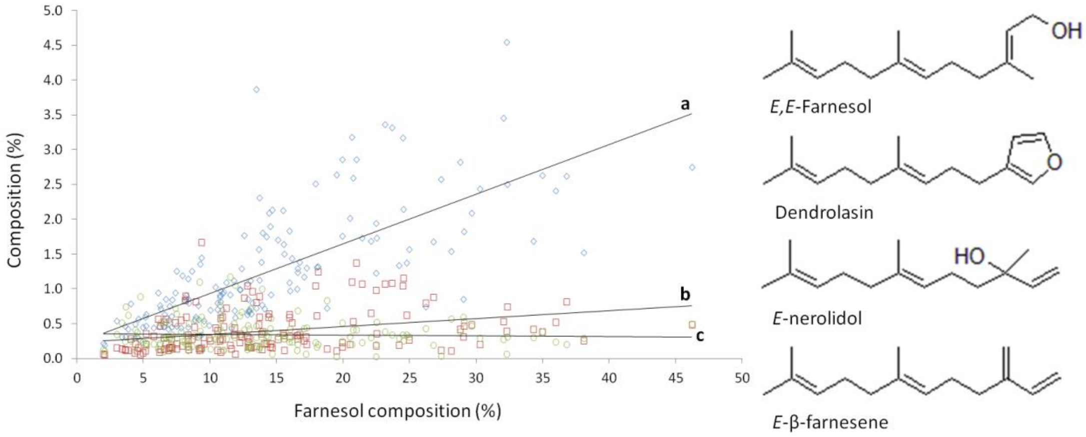

Label	Compound	R^2	P-Value
a	dendrolasin	0.53	$< 2 \times 10^{-16}$
b	*E*-nerolidol	0.1	0.0002467
c	*E*-β-farnesene	0.002	0.0269267

Figure S1. The linear correlation between *E,E*-farnesol, dendrolasin, *E*-nerolidol and *E*-β-farnesene based on GC-MS analysis 194 heartwood cores of *S. spicatum* trees in natural stands of Western Australia: 152 trees from the south-west (Wheatbelt), 19 trees from the south-east (Goldfields) and 23 trees from the north regions (Carnarvon and Shark Bay) using percent composition data.
